# Association of Preterm Birth with Depression and Particulate Matter: Machine Learning Analysis Using National Health Insurance Data

**DOI:** 10.3390/diagnostics11030555

**Published:** 2021-03-19

**Authors:** Kwang-Sig Lee, Hae-In Kim, Ho Yeon Kim, Geum Joon Cho, Soon Cheol Hong, Min Jeong Oh, Hai Joong Kim, Ki Hoon Ahn

**Affiliations:** 1 AI Center, Korea University Anam Hospital, Seoul 02841, Korea; ecophy@hanmail.net (K.-S.L.); radis13@korea.ac.kr (H.-I.K.); 2 School of Industrial Management Engineering, Korea University, Seoul 02841, Korea; 3 Department of Obstetrics & Gynecology, Korea University Anam Hospital, Seoul 02841, Korea; novak082@korea.ac.kr; 4 Department of Obstetrics & Gynecology, Korea University Ansan Hospital, Ansan 15355, Korea; shinbi7873@gmail.com (H.Y.K.); haijkim@korea.ac.kr (H.J.K.); 5 Department of Obstetrics & Gynecology, Korea University Guro Hospital, Seoul 08308, Korea; md_cho@hanmail.net (G.J.C.); mjohmd@korea.ac.kr (M.J.O.)

**Keywords:** preterm birth, particulate matter, depression

## Abstract

This study uses machine learning and population data to analyze major determinants of preterm birth including depression and particulate matter. Retrospective cohort data came from Korea National Health Insurance Service claims data for 405,586 women who were aged 25–40 years and gave births for the first time after a singleton pregnancy during 2015–2017. The dependent variable was preterm birth during 2015–2017 and 90 independent variables were included (demographic/socioeconomic information, particulate matter, disease information, medication history, obstetric information). Random forest variable importance was used to identify major determinants of preterm birth including depression and particulate matter. Based on random forest variable importance, the top 40 determinants of preterm birth during 2015–2017 included socioeconomic status, age, proton pump inhibitor, benzodiazepine, tricyclic antidepressant, sleeping pills, progesterone, gastroesophageal reflux disease (GERD) for the years 2002–2014, particulate matter for the months January–December 2014, region, myoma uteri, diabetes for the years 2013–2014 and depression for the years 2011–2014. In conclusion, preterm birth has strong associations with depression and particulate matter. What is really needed for effective prenatal care is strong intervention for particulate matters together with active counseling and medication for common depressive symptoms (neglected by pregnant women).

## 1. Introduction

Preterm birth is a major part of disease burden for newborns and children on the globe [1,2,3,4]. Every year 15 million babies are born preterm in the world and preterm birth is a main contributor for global neonatal and childhood mortality, i.e., 1 million deaths among those aged 0–4 years [1,2]. For example, one out of every 10 babies was preterm in the United States during 2003–2012, that is, 5,042,982 (12.2%) of 41,206,315 newborns [3]. Indeed, cost-effective interventions are expected to prevent three quarters of mortality from preterm birth [4]. A recent review reports that the following maternal variables are important predictors of preterm birth: demographic/socioeconomic determinants (age, below high school graduation, urban region, insurance, marriage, religion), disease information (delivery/pregestational body mass index, predelivery systolic/diastolic blood pressure, upper gastrointestinal tract symptom, gastroesophageal reflux disease, Helicobacter pylori, gestational diabetes mellitus, systemic lupus erythematosus, increased cerebrospinal fluid and reduced cortical folding due to impaired brain growth), medication history (progesterone, calcium channel blocker, hydroxychloroquine sulfate) and obstetric information (parity, twins, infant sex, prior preterm birth, prior cone biopsy, cervical length, myomas and adenomyosis) [5].

Moreover, emerging literature requests due attention to the significant effects of depression and air pollution on preterm birth [6,7,8,9,10,11,12,13,14,15]. Two systematic reviews reported that prenatal or gestational depression is an important risk factor for preterm birth [6,7]. In addition, two systematic reviews [8,9] and several population-based cohort studies [10,11,12,13,14,15] confirmed a positive association between air pollution and preterm birth. These population-based cohort studies covered various areas and periods including the San Joaquin Valley (the United States, 2000–2006) [10], Ohio (the United States, 2007–2010) [11], Utah (the United States, 2002–2010) [12], Ontario (Canada, 2005–2012) [13], Wuhan (China, 2011–2013) [14] and Korea (2010–2013) [15]. However, the number of predictors in the existing literature has been limited to 14 and no effort has been made based on machine learning in this direction. In this context, this study uses machine learning and population data to analyze major determinants of preterm birth including depression and particulate matter. This study includes a population-based cohort of 405,586 participants and the most comprehensive set of 90 predictors such as demographic/socioeconomic determinants, particulate matter, disease information, medication history and obstetric information.

## 2. Materials and Methods

### 2.1. Participants

Retrospective cohort data for this study came from Korea National Health Insurance Service claims data for 405,586 women, aged 25–40 years who gave birth for the first time after a singleton pregnancy during 2015–2017. South Korea runs a compulsory, universal health insurance service program and Korea National Health Insurance Service claims data cover most health events of all citizens residing in Korea (for more details, visit https://www.nhis.or.kr/static/html/wbd/g/a/wbdga0401.html, accessed on 15 March 2021). This retrospective study was approved by the Institutional Review Board (IRB) of Korea University Anam Hospital on 5 November 2018 (2018AN0365). Informed consent was waived by the Institutional Review Board (IRB) given that data were deidentified.

### 2.2. Variables

The dependent variable was preterm labor and birth during 2015–2017 (birth between 20 weeks and 0 day and 36 weeks and 6 days of gestation). Four categories of preterm labor and birth were defined based on ICD-10 Code: (1) PTB 1—preterm birth with premature rupture of membranes (PROM) only; (2) PTB 2—preterm labor and birth without PROM; (3) PTB 3—PTB 1, PTB 2 or both; (4) PTB 4—PTB 3 or other indicated preterm birth (Supplementary Table S1). This variable was coded as “no” vs. “yes”. The following 90 independent variables were included: (1) demographic/socioeconomic determinants in 2014 such as age (years), socioeconomic status measured by an insurance fee with the range of 1 (the highest group) to 20 (the lowest group), and region (city) (no vs. yes); (2) particulate matter (PM_10_) for each of the months January–December 2014; (3) disease information (no vs. yes) for each of the years 2002–2014, i.e., depression, diabetes, gastroesophageal reflux disease (GERD), hypertension and periodontitis; (4) medication history (no vs. yes) in 2014, i.e., benzodiazepine, calcium channel blocker, nitrate, progesterone, proton pump inhibitor, sleeping pills and tricyclic antidepressant; (5) obstetric information (no vs. yes) in 2014 such as in vitro fertilization, myoma uteri and prior cone. The 65 disease variables were denoted as *Depression_2002, …, Depression_2014, Diabetes_2002, …, Diabetes_2014, GERD_2002, …, GERD_2014, Hypertension_2002, …, Hypertension_2014*, and *Periodontitis_2002, …, Periodontitis_2014*. The disease information and the medication history were screened from ICD-10 and ATC codes, respectively (Supplementary Tables S1 and S2). Indeed, diabetes was defined as fasting glucose equal to or higher than 126 mg/dL or antidiabetic medication. Likewise, hypertension was defined as systolic/diastolic blood pressure equal to or higher than 140/90 mmHg or antihypertensive medication [16]. Finally, particulate matter was denoted as *PM_2014_01* (2014 January)*, …, PM_2014_12* (December 2014) and its monthly average at a district level was obtained from [17]. Introducing the disease and particulate matter variables as above (so called “distributed lag variables”) is one efficient way to analyze the effects of important independent variables in past periods on the dependent variable in the current period.

### 2.3. Analysis

Logistic regression, the random forest and the artificial neural network were applied and compared for the prediction of preterm birth [18]. Data on 402,092 observations with full information were divided into training and validation sets with a 70:30 ratio (281,464 vs. 120,628 observations). Accuracy, a ratio of correct predictions among 120,628 observations, was introduced as a criterion for validating the models trained. Random forest variable importance, which measures the contribution of a variable for the performance of the model, was used for identifying major determinants of preterm birth and testing its associations with depression, particulate matter and other predictors. R-Studio 1.3.959 (R-Studio Inc., Boston, MA, USA) was employed for the analysis during 1 August 2020–31 December 2020.

## 3. Results 

Descriptive statistics for participants’ preterm birth and its determinants are shown in Table 1. Among 405,586 participants, 21,732 (5.40%), 8927 (2.22%), 27,752 (6.90%) and 28,845 (7.17%) belonged to PTB 1, 2, 3 and 4, respectively. The median age and socioeconomic status of the participants were 29 and 12, respectively. Among the participants, 126,008 (31.34%) and 63,066 (15.68%) had proton pump inhibitor and tricyclic antidepressant medications in 2014, respectively. The share of those with depression registered a steady growth from 0.18% in 2002 to 1.36% in 2014. The monthly averages of PM_10_ in Korea’s seven metropolitan areas for the year 2014 were 56 (January), 50 (February), 52 (March), 52 (April), 64 (May), 44 (June), 39 (July), 30 (August), 33 (September), 35 (October), 44 (November) and 42 (December) in terms of 10^−6^ g/m^3^, respectively. In terms of accuracy, the random forest was similar with logistic regression and the artificial neural network (94.50%, 97.66%, 93.08% and 92.83% for PTB 1, PTB 2, PTB 3 and PTB 4 in Table 2, respectively). The results of undersampling are shown in Table 3. Undersampling is an approach to match the sizes of two groups (participants with and without preterm birth) so that the training of machine learning can be balanced between the two groups. Undersampling leads to slight improvement in the performance (the area under the receiver-operating-characteristic curve) of the random forest, e.g., from 0.5585 to 0.5803 in the case of PTB 2.

Based on random forest variable importance, top-40 determinants of preterm birth during 2015–2017 included socioeconomic status, age, proton pump inhibitor, benzodiazepine, tricyclic antidepressant, sleeping pills, progesterone, GERD for the years 2002–2014, particulate matter for the months January2014–December 2014, region, myoma uteri, diabetes for the years 2013–2014 and depression for the years 2011–2014. These values were the averages for PTB 1, PTB 2, PTB 3 and PTB 4 (Supplementary Figure S1 for each of PTB 1, PTB 2, PTB 3 and PTB 4). The importance rankings of particulate matter were particularly high for PTB 2: *PM_2014_08* (5th)*, PM_2014_12* (6th)*, PM_2014_02* (7th)*, PM_2014_11* (8th)*, PM_2014_09* (10th)*, PM_2014_06* (11th)*, PM_2014_10* (12th)*, PM_2014_01* (13th)*, PM_2014_07* (14th)*, PM_2014_05* (15th)*, PM_2014_03* (17th)*, PM_2014_04* (18th). These findings were similar with those of undersampling in Supplementary Figure S2. The results of logistic regression (Table 4 and Table 5) provide useful information about the sign and magnitude for the effect of a major determinant on preterm birth. For example, the odds of PTB 4 will increase by 12.6% if socioeconomic status decreases by 10 in Table 4, e.g., from 2 to 12 (median). The odds of PTB 4 will increase by 24.1% if particulate matter in 2014 August (*PM_2014_08*) increases by 1 × 10^−6^ g/m^3^ in the table. In a similar vein, the odds of PTB 4 will be greater by 12.2% for those with depression in 2010 than those without it in the table.

## 4. Discussion

### 4.1. Findings of This Study

Based on random forest variable importance, top-40 determinants of preterm birth during 2015–2017 included socioeconomic status, age, proton pump inhibitor, benzodiazepine, tricyclic antidepressant, sleeping pills, progesterone, GERD for the years 2002–2014, particulate matter for the months January–December 2014, region, myoma uteri, diabetes for the years 2013–2014 and depression for the years 2011–2014.

### 4.2. Summary of Existing Literature

A recent systematic review reported a positive association between gestational depression and spontaneous preterm labor and birth [6]. This review selected 39 cohort studies with 134,488 participants in total, published in English during 1980–2003. The majority of these studies came from high-income countries such as the United States (27), Denmark (2), France (2), Sweden (2), Canada (1), Norway (1) and the United Kingdom (1). Then, a subsequent systematic review reported that prenatal depression is an important risk factor for preterm birth [7]. This review selected 64 observational studies published in English during 2007 and 2017. Here, 49 (77%) and 15 (23%) of these studies were done in middle-income and low-income countries, respectively. Likewise, two systematic reviews [8,9] stated a positive relationship between air pollution and preterm birth. These reviews selected 15 articles during 1966–2009 and 14 articles during 1995–2012, respectively. These 27 observational or cohort studies were characterized by varying numbers of participants (3853–3,545,777) and diverse origins, i.e., Australia (1), China (2), Canada (4), the Czech Republic (1), Korea (2), Spain (1), the United Kingdom (2) and the United States (14). Their odds-ratio range was 1.05–1.15 regarding PM_2.5_. It would be worthwhile to review several additional population-based cohort studies [10,11,12,13,14,15] on a positive association between air pollution and preterm birth as well. These studies employed 50,005–1,742,183 participants, covering various areas and periods including San Joaquin Valley (the United States, 2000–2006) [10], Ohio (the United States, 2007–2010) [11], Utah (the United States, 2002–2010) [12], Ontario (Canada, 2005–2012) [13], Wuhan (China, 2011–2013) [14] and Korea (2010–2013) [15]. Their odds-ratio ranges were 1.01–1.57 and 1.04–1.19 regarding PM_10_ and PM_2.5_, respectively. However, the number of predictors in the existing literature above has been limited to 14. Moreover, no effort has been made based on machine learning in this line of research.

### 4.3. Contributions of This Study

This study presents the most comprehensive analysis for the determinants of preterm birth, using a population-based cohort of 405,586 participants and the richest collection of 90 predictors such as demographic/socioeconomic determinants, particulate matter, disease information, medication history and obstetric information. Firstly, this study confirms that depression and particulate matter are major predictors of preterm birth (they were the top-40 determinants of preterm birth in this study). Several researchers focus on behavioral, infectious, neuroendocrine and neuroinflammatory mechanisms between depression and preterm birth [19]. Other researchers develop a hypothesis that air pollution causes systemic inflammation, which in turn leads to preterm birth [20]. Little research has been undertaken and more investigation is needed to explore and evaluate various pathways among depression, particulate matter and preterm birth. The findings of this study demonstrate that what is really needed for effective prenatal care is strong intervention for particulate matter together with active counseling and medication for common depressive symptoms (neglected by pregnant women). Secondly, the results of this study agree with those of a previous study with 731 participants on gastroesophageal reflux disease, medication history and preterm birth [18]: The findings of this previous study highlighted the significance of age, socioeconomic status (below high school graduation), progesterone medication history, gastroesophageal reflux disease, region (city) and gestational diabetes mellitus. Above all, to the best of our knowledge, this study is the first attempt to use machine learning and population data to find the main predictors of preterm birth and evaluate its association with depression and particulate matter. This study will be a good starting point in this direction to find main predictors of preterm birth and draw effective implications for its prevention and management.

### 4.4. Limitations of This Study

Firstly, this study did not examine possible mediating effects among variables. Secondly, this study adopted the binary category of preterm birth as no vs. yes (birth between 20 weeks and 0 day and 36 weeks and 6 days of gestation). But preterm birth can have multiple categories and it will be a good topic for future study to compare different predictors for various categories of preterm birth, e.g., extremely preterm (less than 28 (or 24) weeks), very preterm (28–32 (or 24–32) weeks), moderate to late preterm (32–37 weeks) [2]. Thirdly, four categories of preterm birth were defined based on the ICD-10 Code and this could be a source of potential bias. Fourthly, it was not the scope of this study to explore and evaluate various pathways among depression, particulate matter and preterm birth. Little research has been undertaken and more investigations are needed on this topic. Fifthly, uniting various kinds of deep learning approaches for various kinds of preterm birth data would bring new innovations and deeper insights in this line of research. Finally, further investigations of single vs. multiple gestation would deliver more insights and more detailed clinical implications.

### 4.5. Conclusions

Preterm birth has strong associations with depression and particulate matter. What is really needed for effective prenatal care is strong intervention for particulate matters together with active counseling and medication for common depressive symptoms (neglected by pregnant women).

## Figures and Tables

**Table 1:** Descriptive statistics on preterm birth and its determinants.

Variable	No	Yes	Yes (%)
PTB 1 ^a^	380,360	21,732	5.40
PTB 2	393,165	8927	2.22
PTB 3	374,340	27,752	6.90
PTB 4	373,247	28,845	7.17
Benzodiazepine	165,773	236,319	58.77
Calcium Channel Blocker	398,352	3740	0.93
Diabetes_2002	401,226	866	0.22
Diabetes_2003	401,079	1013	0.25
Diabetes_2004	400,833	1259	0.31
Diabetes_2005	400,306	1786	0.44
Diabetes_2006	400,348	1744	0.43
Diabetes_2007	400,302	1790	0.45
Diabetes_2008	400,211	1881	0.47
Diabetes_2009	400,062	2030	0.50
Diabetes_2010	399,833	2259	0.56
Diabetes_2011	399,491	2601	0.65
Diabetes_2012	399,027	3065	0.76
Diabetes_2013	398,048	4044	1.01
Diabetes_2014	395,699	6393	1.59
Depression_2002	400,551	727	0.18
Depression_2003	400,328	950	0.24
Depression_2004	400,068	1210	0.30
Depression_2005	399,467	1811	0.45
Depression_2006	399,112	2166	0.54
Depression_2007	398,494	2784	0.69
Depression_2008	398277	3001	0.75
Depression_2009	397,877	3401	0.85
Depression_2010	397,422	3856	0.96
Depression_2011	396,951	4327	1.08
Depression_2012	395,929	5349	1.33
Depression_2013	395,971	5307	1.32
Depression_2014	395,837	5441	1.36
GERD_2002 ^b^	399,076	3016	0.75
GERD_2003	398,129	3963	0.99
GERD_2004	396,932	5160	1.28
GERD_2005	395,351	6741	1.68
GERD_2006	393,244	8848	2.20
GERD_2007	389,177	12,915	3.21
GERD_2008	386,219	15,873	3.95
GERD_2009	380,452	21,640	5.38
GERD_2010	376,619	25,473	6.34
GERD_2011	372,819	29,273	7.28
GERD_2012	368,833	33,259	8.27
GERD_2013	367,240	34,852	8.67
GERD_2014	363,411	38,681	9.62
Hypertension_2002	401,492	600	0.15
Hypertension_2003	401,464	628	0.16
Hypertension_2004	401,360	732	0.18
Hypertension_2005	401,196	896	0.22
Hypertension_2006	401,088	1004	0.25
Hypertension_2007	400,968	1124	0.28
Hypertension_2008	400,844	1248	0.31
Hypertension_2009	400,718	1374	0.34
Hypertension_2010	400,714	1378	0.34
Hypertension_2011	400,738	1354	0.34
Hypertension_2012	400,406	1686	0.42
Hypertension_2013	400,187	1905	0.47
Hypertension_2014	399,850	2242	0.56
In Vitro Fertilization	401,965	127	0.03
Myoma Uteri	385,015	17,077	4.25
Nitrate	400,776	1316	0.33
Periodontitis_2002	401,895	197	0.05
Periodontitis_2003	401,830	262	0.07
Periodontitis_2004	401,688	404	0.10
Periodontitis_2005	401,665	427	0.11
Periodontitis_2006	401,502	590	0.15
Periodontitis_2007	401,783	309	0.08
Periodontitis_2008	401,795	297	0.07
Periodontitis_2009	401,742	350	0.09
Periodontitis_2010	401,753	339	0.08
Periodontitis_2011	401,797	295	0.07
Periodontitis_2012	401,837	255	0.06
Periodontitis_2013	401,824	268	0.07
Periodontitis_2014	401,854	238	0.06
Prior Cone	401,911	181	0.05
Progesterone	307,684	94,408	23.48
Proton Pump Inhibitor	276,084	126,008	31.34
Region (City)	28,615	373,477	92.88
Sleeping Pills	370,303	31,789	7.91
Tricyclic Antidepressant	339,026	63,066	15.68

^a^ PTB, preterm birth during 2015–2017; ^b^ GERD, gastroesophageal reflux disease.

**Table 2:** Model performance.

Model	Accuracy				AUC ^a^			
	PTB 1 ^b^	PTB 2	PTB 3	PTB 4	PTB 1	PTB 2	PTB 3	PTB 4
Logistic Regression	0.9450	0.9766	0.9308	0.9283	0.5536	0.5916	0.5599	0.5610
Artificial Neural Network	0.9450	0.9766	0.9308	0.9283	0.5000	0.5000	0.5000	0.5000
Random Forest	0.9450	0.9766	0.9308	0.9283	0.5275	0.5585	0.5407	0.5407

^a^ Area under the receiver-operating-characteristic curve; ^b^ PTB, preterm birth during 2015–2017.

**Table 3:** Model performance with undersampling.

Model	Accuracy				AUC ^a^			
	PTB 1 ^b^	PTB 2	PTB 3	PTB 4	PTB 1	PTB 2	PTB 3	PTB 4
Logistic Regression	0.9448	0.9691	0.9302	0.9276	0.5550	0.5872	0.5567	0.5621
Artificial Neural Network	0.9450	0.9766	0.9308	0.9283	0.5000	0.5000	0.5000	0.5000
Random Forest	0.9399	0.9550	0.9251	0.9218	0.5535	0.5803	0.5517	0.5601

^a^ Area under the receiver-operating-characteristic curve; ^b^ PTB, preterm birth during 2015–2017.

**Table 4:** Coefficients of determinants from logistic regression for each type of preterm birth.

Determinant	PTB 1 ^a^	PTB 2	PTB 3	PTB 4
Age	** 1.0000	** 1.0000	1.0000	** 1.0000
Benzodiazepine	** 1.0004	** 1.6725	** 1.0034	** 1.0017
Calcium Channel Blocker	* 1.6383	1.5038	* 1.1644	1.0681
Diabetes_2002	1.8353	2.5656	2.3002	1.5692
Diabetes_2003	1.9481	2.0809	1.2222	1.1303
Diabetes_2004	2.4480	1.6662	1.9147	1.7558
Diabetes_2005	1.0359	** 1.5251	1.0404	** 1.683
Diabetes_2006	1.5526	1.5065	1.2636	2.0387
Diabetes_2007	1.7720	1.1203	2.4172	1.3727
Diabetes_2008	1.9223	2.3487	1.1073	2.5957
Diabetes_2009	1.3161	2.0164	1.0789	*1.2377
Diabetes_2010	1.8100	1.2418	1.7370	1.4228
Diabetes_2011	1.2535	1.2649	2.3813	1.4582
Diabetes_2012	1.5008	1.8574	1.4972	1.1276
Diabetes_2013	2.5368	1.5377	1.7506	2.0177
Diabetes_2014	** 1.0077	** 1.0000	1.0000	** 1.0000
Depression_2002	1.5841	1.0117	** 2.1808	2.2863
Depression_2003	1.0729	* 1.1167	2.0087	1.8181
Depression_2004	1.8705	1.0978	*1.8549	2.0803
Depression_2005	1.1511	1.3906	2.4433	1.5790
Depression_2006	1.0631	* 1.8224	1.1180	1.4364
Depression_2007	1.0956	* 1.7598	1.3947	1.8300
Depression_2008	2.4007	1.3747	1.5346	2.4277
Depression_2009	2.1026	2.1431	2.5796	2.0482
Depression_2010	1.0200	** 2.6590	1.0441	** 1.1220
Depression_2011	2.4402	2.0030	1.9169	2.1260
Depression_2012	1.7774	2.6371	1.9263	1.3044
Depression_2013	** 1.6356	2.0957	1.0422	1.0106
Depression_2014	1.2603	1.6195	** 1.4356	1.2745
GERD_2002	1.3563	1.9083	1.2087	1.1254
GERD_2003	1.1052	2.0605	1.0871	*1.1697
GERD_2004	2.2782	1.5084	1.2115	1.2032
GERD_2005	** 1.4589	1.1591	1.0435	** 1.0084
GERD_2006	2.3188	1.7606	2.4122	1.6032
GERD_2007	1.1257	1.9426	1.5393	1.3863
GERD_2008	2.0329	2.0811	1.2242	1.2627
GERD_2009	1.0689	* 1.1254	1.5055	1.9012
GERD_2010	1.1983	1.5844	1.8026	2.5059
GERD_2011	** 1.1285	2.4433	1.1078	1.0503
GERD_2012	1.2868	1.0417	** 1.311	1.1167
GERD_2013	2.1698	1.7186	2.5039	2.2581
GERD_2014	2.0394	1.4160	1.3279	1.5451
Hypertension_2002	1.0275	** 1.5738	1.0272	** 1.1747
Hypertension_2003	1.1702	1.5249	2.4853	2.5978
Hypertension_2004	1.0521	*2.4381	1.7597	1.1924
Hypertension_2005	1.7869	1.5763	1.503	2.2755
Hypertension_2006	** 1.0203	** 2.1423	1.0638	* 1.0457
Hypertension_2007	2.4269	1.201	2.2096	2.3402
Hypertension_2008	1.3228	1.2716	1.2146	1.2731
Hypertension_2009	1.2009	1.6697	2.1321	1.6487
Hypertension_2010	1.0225	** 1.0764	* 1.0271	** 1.0418
Hypertension_2011	1.9046	1.2264	1.0831	*2.3515
Hypertension_2012	1.2585	2.5826	2.4341	2.2499
Hypertension_2013	2.4772	1.0218	** 1.7588	1.8997
Hypertension_2014	** 1.3136	1.0142	** 1.0008	** 1.0227
In Vitro Fertilization	** 1.3427	1.0005	** 1.0120	** 1.0002
Myoma Uteri	** 1.0000	** 1.0000	** 1.0000	** 1.0000
Nitrate	1.9893	1.7917	1.9809	1.4776
Periodontitis_2002	1.9718	2.0034	1.3465	2.0526
Periodontitis_2003	1.4198	1.7779	1.4892	1.9032
Periodontitis_2004	** 1.1103	1.8267	1.2493	1.0187
Periodontitis_2005	1.2443	1.2035	1.6319	2.5775
Periodontitis_2006	** 1.3461	1.0005	** 1.4314	1.0181
Periodontitis_2007	1.3134	1.7419	1.6292	1.7522
Periodontitis_2008	1.8989	2.0237	2.1047	1.7572
Periodontitis_2009	1.3065	1.6335	2.2664	2.4738
Periodontitis_2010	2.0266	2.3908	1.0987	* 2.1828
Periodontitis_2011	1.3008	1.7355	1.6395	2.6100
Periodontitis_2012	2.4308	1.3264	1.1636	1.7730
Periodontitis_2013	1.1598	2.5870	1.2379	1.2664
Periodontitis_2014	1.3858	1.5461	1.5541	1.2876
PM_2014_01	** 1.0000	** 1.0639	** 1.0001	** 1.0002
PM_2014_02	** 1.0000	** 1.0483	** 1.0000	** 1.0000
PM_2014_03	** 1.0214	** 1.0028	** 1.0000	** 1.0003
PM_2014_04	1.9584	1.0355	** 1.0887	*1.1566
PM_2014_05	** 1.0032	** 1.0006	** 1.0005	** 1.0004
PM_2014_06	** 1.0103	** 1.0000	1.0008	** 1.0000
PM_2014_07	** 1.0000	** 2.0301	1.0000	** 1.0000
PM_2014_08	2.5322	1.2738	1.1038	*1.2412
PM_2014_09	** 1.0001	** 1.1734	1.0059	** 1.0001
PM_2014_10	** 1.0020	** 2.6945	1.0041	** 1.0007
PM_2014_11	1.7164	1.4224	1.7286	1.2650
PM_2014_12	** 1.0631	* 1.5461	1.0958	* 1.0325
Prior Cone	1.1881	1.7899	2.5560	2.1212
Progesterone	** 1.0000	** 1.0000	** 1.0000	** 1.0000
Proton Pump Inhibitor	* 1.0314	** 1.8051	1.1315	1.0870
Region (City)	* 1.0000	** 1.0068	** 1.3983	1.0564
Sleeping Pills	1.1783	1.4780	1.6950	1.9664
Socioeconomic Status	** 1.6547	1.5079	1.0856	* 1.0126
Tricyclic Antidepressant	** 1.0065	** 1.3169	1.0613	* 1.0223

^a^ PTB, preterm birth during 2015–2017; * *p < 0.10*, ** *p < 0.05.*

**Table 5:** Coefficients of determinants from logistic regression with undersampling.

Determinant	PTB 1 ^a^	PTB 2	PTB 3	PTB 4
Age	** 1.0000	** 1.0000	1.0000	** 1.0000
Benzodiazepine	** 1.0317	1.5222	** 1.0198	1.3658
Calcium Channel Blocker	2.0962	1.1379	1.6327	** 1.0065
Diabetes_2002	2.4011	1.5789	1.4489	1.7523
Diabetes_2003	1.1577	** 1.0046	1.6649	2.2509
Diabetes_2004	1.2701	1.4161	1.2806	2.0063
Diabetes_2005	** 1.0306	1.6731	1.6038	** 1.0338
Diabetes_2006	1.7435	2.5349	1.8879	1.9066
Diabetes_2007	2.0364	1.5802	1.4317	2.2514
Diabetes_2008	2.5007	1.8168	2.1137	1.3937
Diabetes_2009	2.0164	** 1.0272	2.3441	* 1.0568
Diabetes_2010	*1.0748	2.0294	* 1.0843	1.9013
Diabetes_2011	1.4167	2.6692	1.3705	1.6768
Diabetes_2012	2.0074	1.4708	* 1.0748	1.4180
Diabetes_2013	2.6481	1.4699	1.3525	1.5072
Diabetes_2014	** 1.0004	** 1.0000	** 1.0000	** 1.0000
Depression_2002	1.9737	2.2064	1.9423	2.0489
Depression_2003	1.6566	1.2687	1.506	1.2128
Depression_2004	1.5461	1.4561	1.1245	2.2313
Depression_2005	2.1117	2.2828	1.3139	* 1.0695
Depression_2006	1.4251	1.6441	1.6534	** 1.0422
Depression_2007	1.4740	2.0573	** 1.0505	1.9812
Depression_2008	1.3009	1.7328	2.2586	** 1.0480
Depression_2009	2.5888	2.5528	1.3725	2.2751
Depression_2010	1.2970	1.6665	1.4457	* 1.0910
Depression_2011	2.5129	1.7514	1.4860	1.5786
Depression_2012	1.4842	2.0179	1.5231	1.3482
Depression_2013	1.1655	1.4877	** 1.0437	** 1.0402
Depression_2014	1.2937	1.1992	1.8507	1.6490
GERD_2002	2.7067	2.5489	1.2727	2.2506
GERD_2003	2.0535	* 1.0868	** 1.0511	1.2279
GERD_2004	1.7188	1.1785	1.6052	1.2509
GERD_2005	1.4594	2.0748	1.1597	** 1.0127
GERD_2006	2.3703	2.2252	1.7161	2.4705
GERD_2007	1.7442	1.3426	2.0336	2.5701
GERD_2008	1.9237	1.7541	1.6918	1.3901
GERD_2009	1.2527	1.3282	1.5109	2.5045
GERD_2010	2.0095	1.7975	1.7073	1.2688
GERD_2011	** 1.0214	1.3559	** 1.0440	1.4040
GERD_2012	2.3677	1.4217	1.2529	1.9972
GERD_2013	2.0424	1.4997	1.5437	1.6973
GERD_2014	1.2615	1.7767	*1.0782	1.2339
Hypertension_2002	1.1105	1.5397	*1.0819	*1.0678
Hypertension_2003	1.2966	1.2001	2.7089	1.7429
Hypertension_2004	** 1.0265	1.3399	2.4372	1.1225
Hypertension_2005	1.8251	1.3401	1.5551	2.3208
Hypertension_2006	* 1.0748	1.9067	1.3699	1.1140
Hypertension_2007	2.3224	2.6096	2.0325	1.1384
Hypertension_2008	1.1152	1.9666	1.3094	1.6447
Hypertension_2009	2.0016	2.6371	1.3935	1.4603
Hypertension_2010	1.5264	1.7342	* 1.0928	** 1.0022
Hypertension_2011	1.3179	2.2948	1.3472	2.2883
Hypertension_2012	1.4354	1.2743	1.6905	1.6833
Hypertension_2013	1.7502	1.2018	* 1.068	1.3578
Hypertension_2014	1.8333	** 1.0064	1.3089	1.7927
In Vitro Fertilization	1.2642	1.1235	* 1.0986	1.7811
Myoma Uteri	** 1.0000	** 1.0000	** 1.0000	** 1.0000
Nitrate	2.3581	*1.0525	2.1427	2.2613
Periodontitis_2002	2.2901	1.2350	1.1919	1.5518
Periodontitis_2003	2.3085	1.1780	1.7492	1.7112
Periodontitis_2004	* 1.0720	1.1799	1.4031	*1.0831
Periodontitis_2005	2.5496	** 1.0224	1.8663	1.3206
Periodontitis_2006	1.1120	* 1.0665	1.1650	1.2683
Periodontitis_2007	2.1555	1.3009	2.3910	2.2307
Periodontitis_2008	1.7323	2.1782	1.2830	1.4805
Periodontitis_2009	* 1.0585	1.9502	1.2883	1.1346
Periodontitis_2010	2.2747	2.5854	* 1.0989	1.4304
Periodontitis_2011	2.1601	1.8406	1.6396	2.6411
Periodontitis_2012	2.4818	1.6958	2.2394	1.3557
Periodontitis_2013	1.6737	1.5204	1.8702	1.1657
Periodontitis_2014	1.9485	2.1991	1.2673	* 1.0691
PM_2014_01	** 1.0001	1.7761	** 1.0000	** 1.0052
PM_2014_02	** 1.0000	** 1.0192	** 1.0000	** 1.0000
PM_2014_03	1.1123	** 1.0092	** 1.0002	** 1.0000
PM_2014_04	1.5234	2.4338	1.1769	1.3751
PM_2014_05	** 1.0005	** 1.0099	** 1.0047	** 1.0011
PM_2014_06	** 1.0002	** 1.0028	** 1.0059	** 1.0002
PM_2014_07	** 1.0006	2.6328	** 1.0013	** 1.0003
PM_2014_08	1.1148	* 1.0980	2.6242	1.3607
PM_2014_09	** 1.0000	** 1.0053	** 1.0075	** 1.0340
PM_2014_10	** 1.0004	* 1.0860	** 1.0042	* 1.0966
PM_2014_11	1.1574	1.8433	1.7954	1.5836
PM_2014_12	** 1.0367	1.4980	1.3242	1.2909
Prior Cone	1.3822	2.1422	1.3633	1.1997
Progesterone	** 1.0000	** 1.0000	** 1.0000	** 1.0000
Proton Pump Inhibitor	*1.0945	2.3852	1.1117	1.1141
Region (City)	** 1.0000	1.3816	1.2491	1.1881
Sleeping Pills	1.1788	1.1526	2.6498	1.9911
Socioeconomic Status	** 1.0314	1.2623	1.2843	** 1.0041
Tricyclic Antidepressant	** 1.0003	2.5491	1.3516	** 1.0188

^a^ PTB, preterm birth during 2015–2017; * *p < 0.10*, ** *p < 0.05.*

## Data Availability

The data presented in this study are not publicly available. But the data are available from the corresponding author upon reasonable request and under the permission of Korea National Health Insurance Service.

## Supplementary Materials

The following are available online at https://www.mdpi.com/2075-4418/11/3/555/s1, Figure S1: Random forest variable importance, Figure S2: Random forest variable importance—undersampling, Table S1: ICD-10 code for preterm birth, depression, gastroesophageal reflux disease and periodontitis, Table S2: ATC code for medication.
