# Supplementary material for: Association of Preterm Birth with Depression and Particulate Matter: Machine Learning Analysis Using National Health Insurance Data
Source: Diagnostics (Basel). 2021 Mar 19;11(3):555. doi: 10.3390/diagnostics11030555 (PMC8003604; doi:10.3390/diagnostics11030555)
Supplement: Supplementary file 1 [file diagnostics-11-00555-s001.pdf]

Supplementary Materials

Association of Preterm Birth with Depression and Particulate Matter: Machine Learning Analysis Using National Health Insurance Data

Average

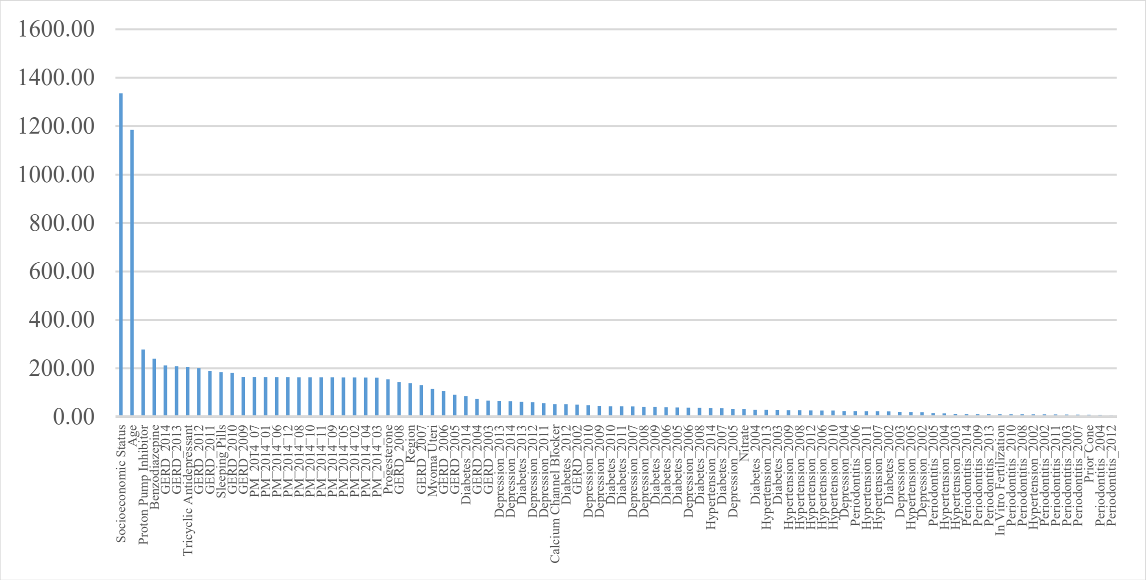

PTB1

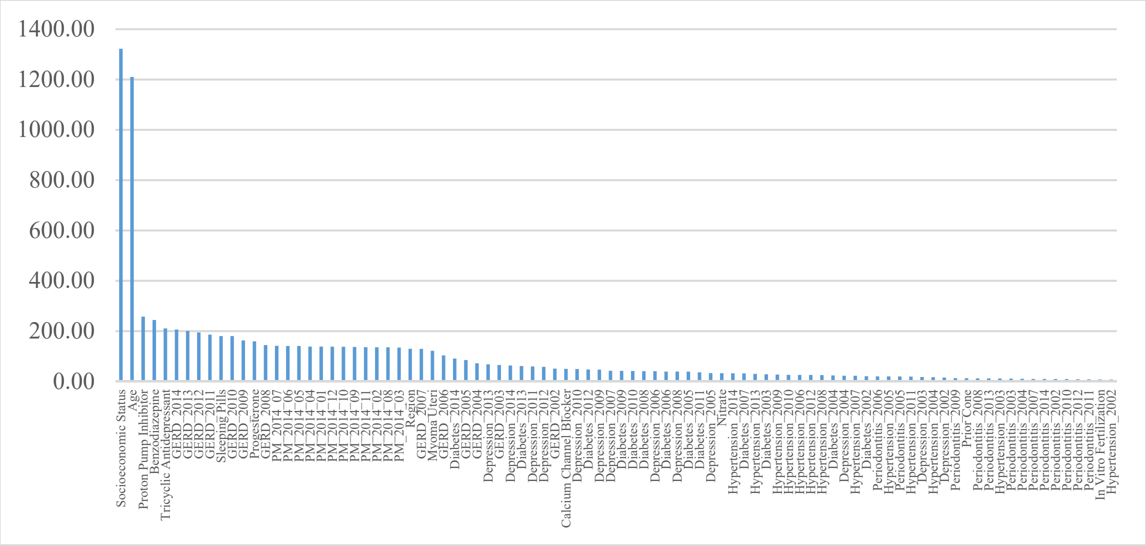

PTB2

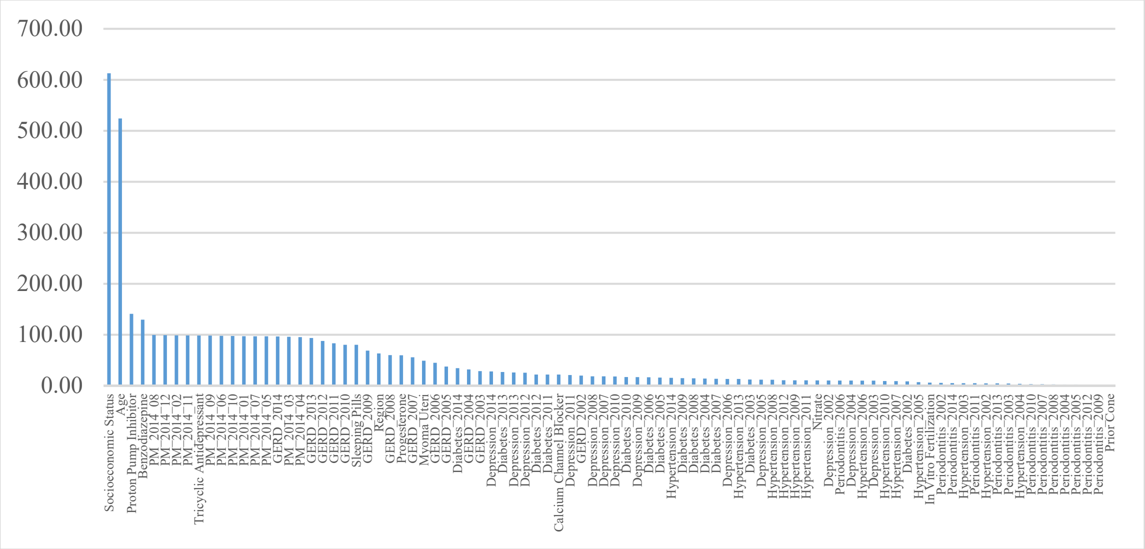

PTB3

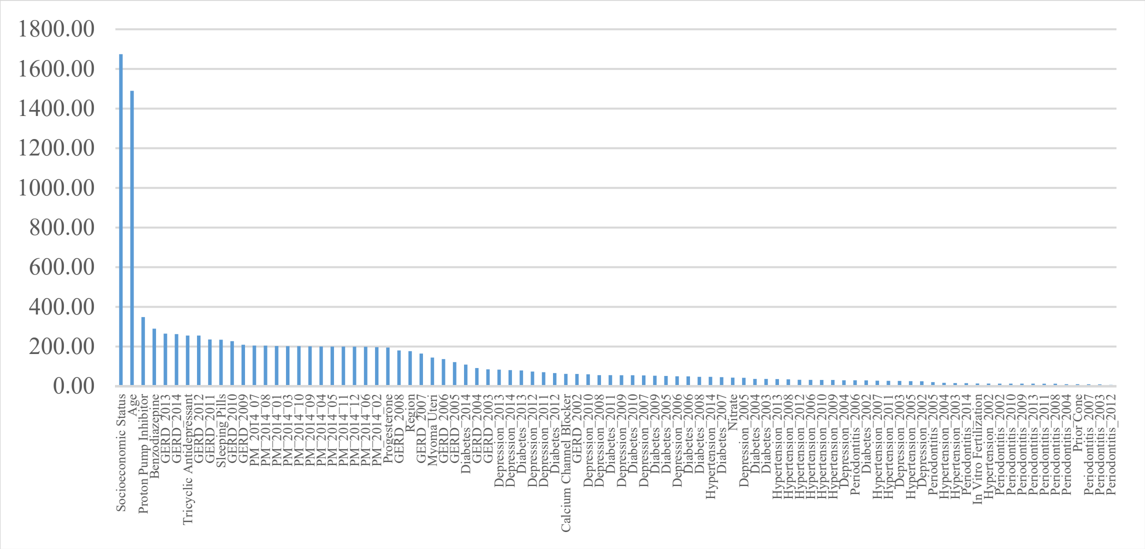

PTB4

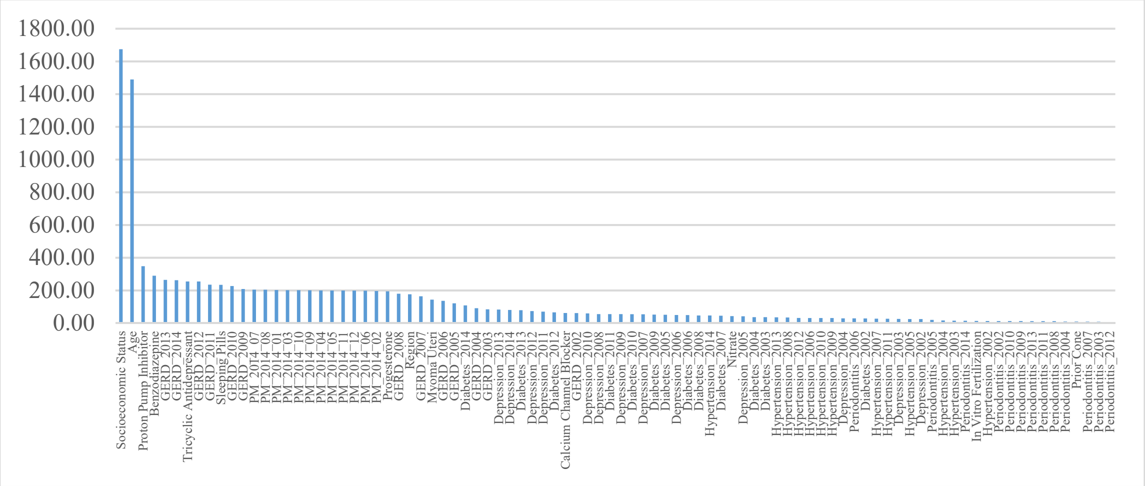

Figure S1. Random forest variable importance.

Average

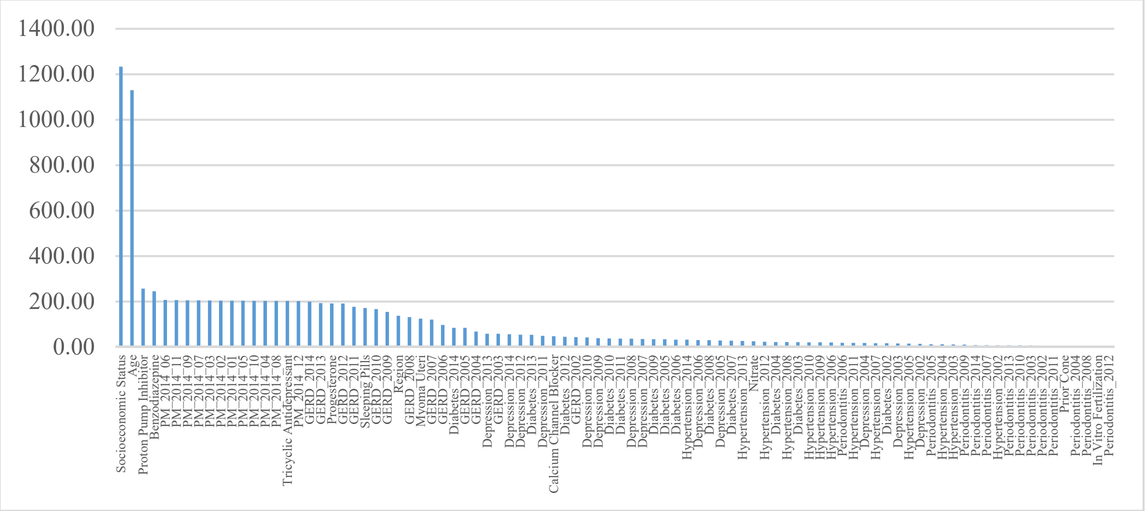

PTB1

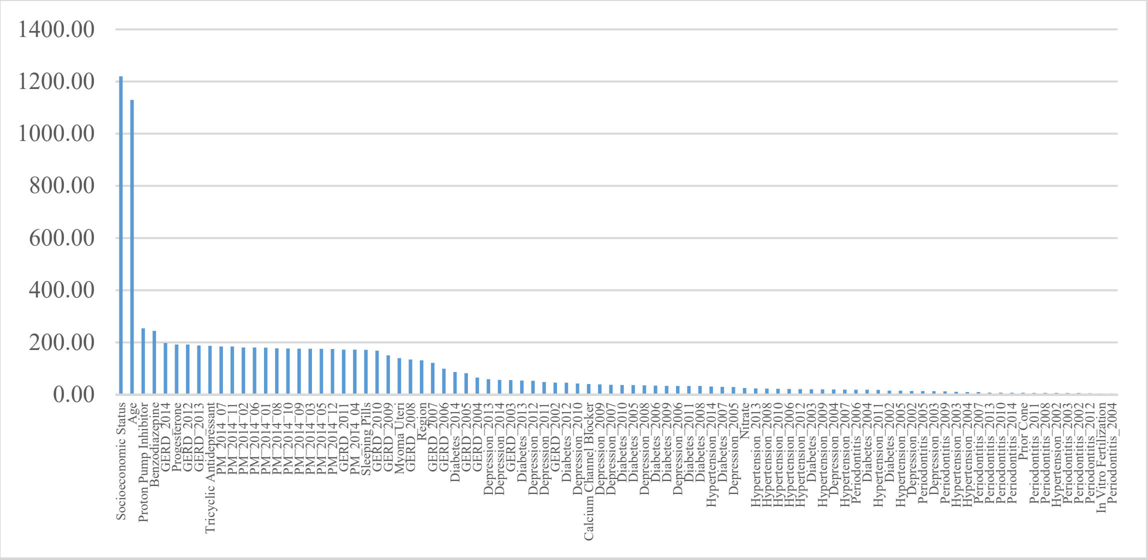

PTB2

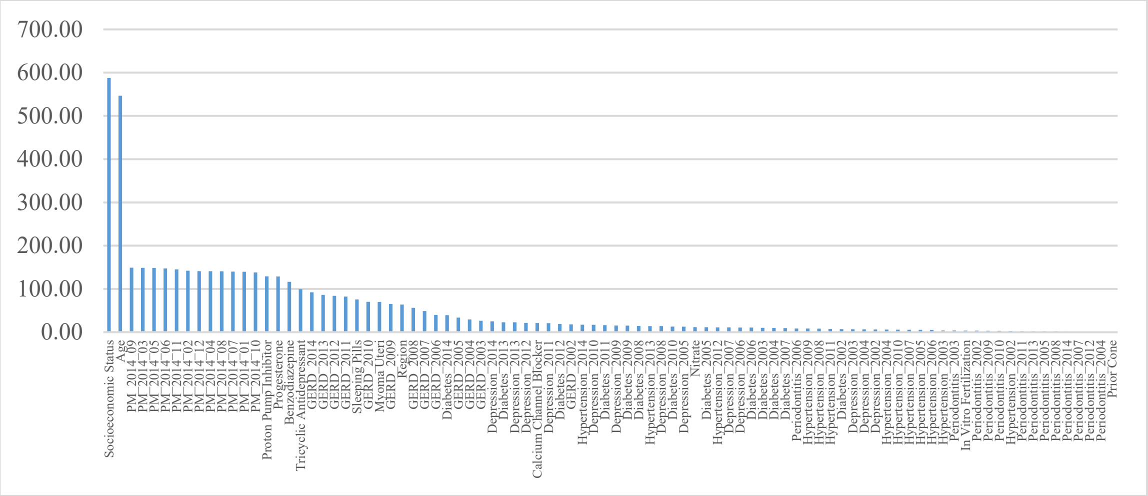

PTB3

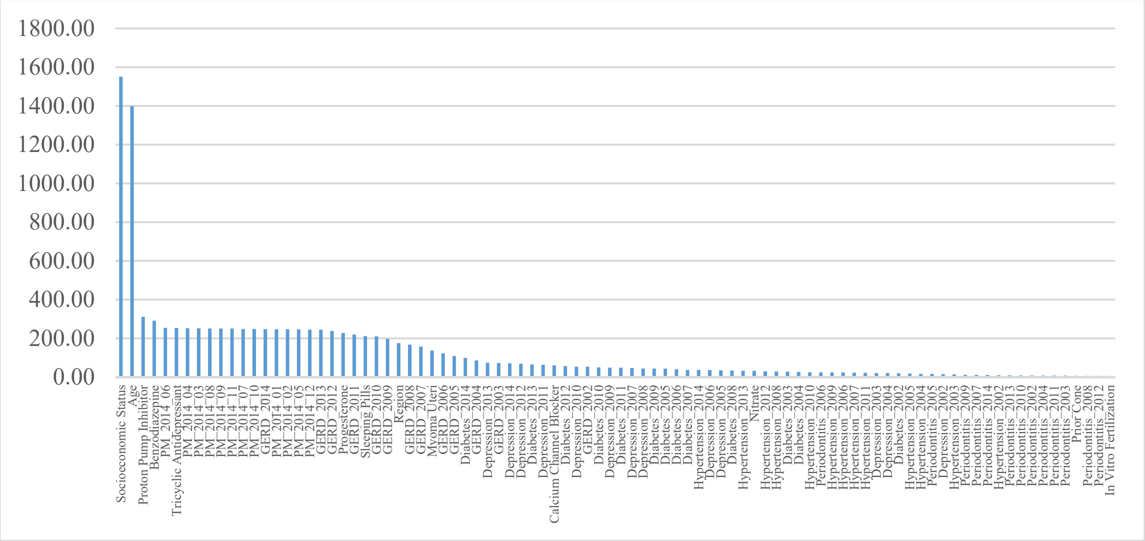

PTB4

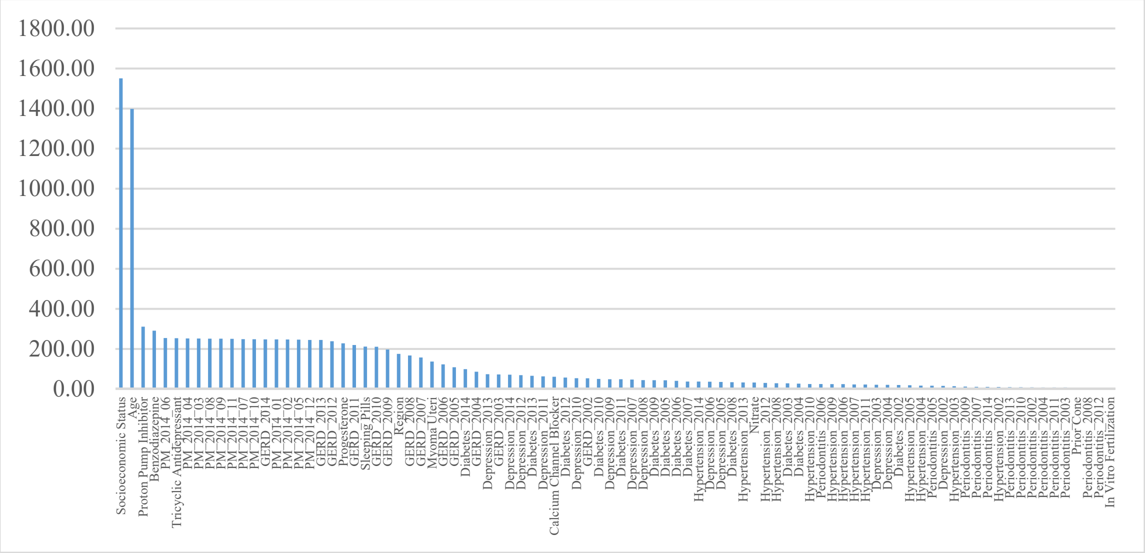

Figure S2. Random forest variable importance—undersampling.

Table S1. ICD-10 code for preterm birth, depression, gastroesophageal reflux disease and periodontitis.

		Code	Description
PTB	PROM	O42.00	Onset of labor within 24 hours of rupture (0–33 weeks of gestation)
		O42.01	Onset of labor within 24 hours of rupture (34–36 weeks of gestation)
		O42.10	Onset of labor after 24 hours of rupture (0–33 weeks of gestation)
		O42.11	Onset of labor after 24 hours of rupture (34–36 weeks of gestation)
		O42.20	Prolonged labor because of treatment (0–33 weeks of gestation)
		O42.21	Prolonged labor because of treatment (34–36 weeks of gestation)
		O42.90	Unspecified length of time between rupture and onset of labor (0–33 weeks of gestation)
		O42.91	Unspecified length of time between rupture and onset of labor (34–36 weeks of gestation)
	PTL	O60.11	Preterm delivery with preterm labor (0–33 weeks of gestation)
		O60.12	Preterm delivery with preterm labor (34–36 weeks of gestation)
		O60.19	Preterm delivery with preterm labor (unspecified weeks of gestation)
	Other	O60.31	Preterm delivery without preterm labor (0–33 weeks of gestation)
		O60.32	Preterm delivery without preterm labor (34–36 weeks of gestation)
		O60.39	Preterm delivery without preterm labor (unspecified weeks of gestation)
Depression		F32	Depressive episode
GERD		K210	Gastroesophageal reflux disease with esophagitis
Periodontitis		K052	Aggressive periodontitis
		K0528	Other aggressive periodontitis
		K0529	Unspecified aggressive periodontitis
		K053	Chronic periodontitis
		K0538	Other chronic periodontitis
		K0539	Unspecified chronic periodontitis

PTB Preterm Birth, PROM Premature Rupture of Membranes, PTL Preterm Labor with Preterm Delivery, GERD Gastroesophageal Reflux Disease.

Table S2. ATC code for medication.

Medication	Code	Description	
Benzodiazepine	N05BA	Benzodiazepine derivatives	(N05 Psychoanaleptics, N05B Anxiolytics)
	N05CD	Benzodiazepine derivatives	(N05 Psychoanaleptics, N05C Hypnotics and sedatives)
	N05CF	Benzodiazepine-related drugs	(N05 Psychoanaleptics, N05C Hypnotics and sedatives)
Calcium Channel Blocker	C08	Calcium channel blockers	
Nitrate	C01DA	Organic nitrates	
Progesterone	G03	Sex hormones and modulators of the genital system	
Proton Pump Inhibitor	A02BC	Proton pump inhibitors	
Sleeping Pills	N05C	Hypnotics and sedatives	
Tricyclic Antidepressant	N06A	Antidepressants	
